# Effect of vestibular exercise and optokinetic stimulation using virtual reality in persistent postural-perceptual dizziness

**DOI:** 10.1038/s41598-021-93940-z

**Published:** 2021-07-14

**Authors:** Seo-Young Choi, Jae-Hwan Choi, Eun Hye Oh, Se-Joon Oh, Kwang-Dong Choi

**Affiliations:** 1 Department of Neurology, College of Medicine, Pusan National University Hospital, Pusan National University School of Medicine and Biomedical Research Institute, 179, Gudeok-ro, Seo-gu, Busan, 602-739 Korea; 2 Department of Neurology, Pusan National University School of Medicine, Research Institute for Convergence of Biomedical Science and Technology, Pusan National University Yangsan Hospital, Busan, Korea; 3 Department of Otorhinolaryngology and Biomedical Research Institute, Pusan National University Hospital, Busan, Korea

**Keywords:** Diseases, Neurology

## Abstract

To determine the effect of customized vestibular exercise (VE) and optokinetic stimulation (OS) using a virtual reality system in patients with persistent postural-perceptual dizziness (PPPD). Patients diagnosed with PPPD were randomly assigned to the VE group or VE with OS group. All participants received VE for 20 min using a virtual reality system with a head mount display once a week for 4 weeks. The patients in the VE with OS group additionally received OS for 9 min. We analysed the questionnaires, timed up-to-go (TUG) test, and posturography scores at baseline and after 4 weeks. A total of 28 patients (median age = 74.5, IQR 66–78, men = 12) completed the intervention. From baseline to 4 weeks, the dizziness handicap inventory, activities of daily living (ADL), visual vertigo analogue scale, and TUG improved in the VE group, but only ADL and TUG improved in the VE with OS group. Patients with severe visual vertigo improved more on their symptoms than patients with lesser visual vertigo (Pearson’s p = 0.716, *p* < 0.001). Our VE program can improve dizziness, quality of life, and gait function in PPPD; however, additional optokinetic stimuli should be applied for individuals with visual vertigo symptoms.

## Introduction

Persistent postural-perceptual dizziness (PPPD) is a recently defined diagnostic syndrome that unifies key features of chronic subjective dizziness, visual vertigo, phobic postural vertigo, and related disorders^1,2^. Because PPPD is a chronic functional and portmanteau disorder of the nervous system that presents as dizziness or imbalance due to a neurologic and a medical condition and coupled with psychological distress, a multidisciplinary approach for treatment should be considered^3^. Medications such as selective serotonin reuptake inhibitors or serotonin-norepinephrine reuptake inhibitors, cognitive behavioural psychotherapy, and/or vestibular rehabilitation therapy (VRT) are generally recommended in PPPD^4^.

The theoretical basis for VRT is sensory reweighing, which is the redistribution of vision, somatosensation, and vestibular sense for balance^5^, but few studies have been conducted to determine whether specific exercises should be designed to augment an individual’s other sensory strength or to boost vestibular weakness^6^. In particular, because VRT includes not only compensation training after vestibular loss but also postural exercises in other causes of dizziness or general unsteadiness^6^, it can be applicable to patients with PPPD, which is entwined by variable pathophysiology^3^.

Among the sensory components of VRT, visual stimuli using virtual reality environments are not only manoeuvrable but also effective in patients with dizziness and visual vertigo symptoms^7,8^. Because visual vertigo is a closely related symptom of PPPD^2^, specific vestibular exercise focused on visual stimuli can be an effective treatment in PPPD; however, few randomized controlled trials on the efficacy of VRT in PPPD have been reported.

We conducted a randomized controlled trial to determine the short-term efficacy of customized vestibular exercise (VE) and optokinetic stimulation (OS) using a mobile virtual reality system in patients with PPPD.

## Results

### Characteristics at baseline

**Figure 1:**
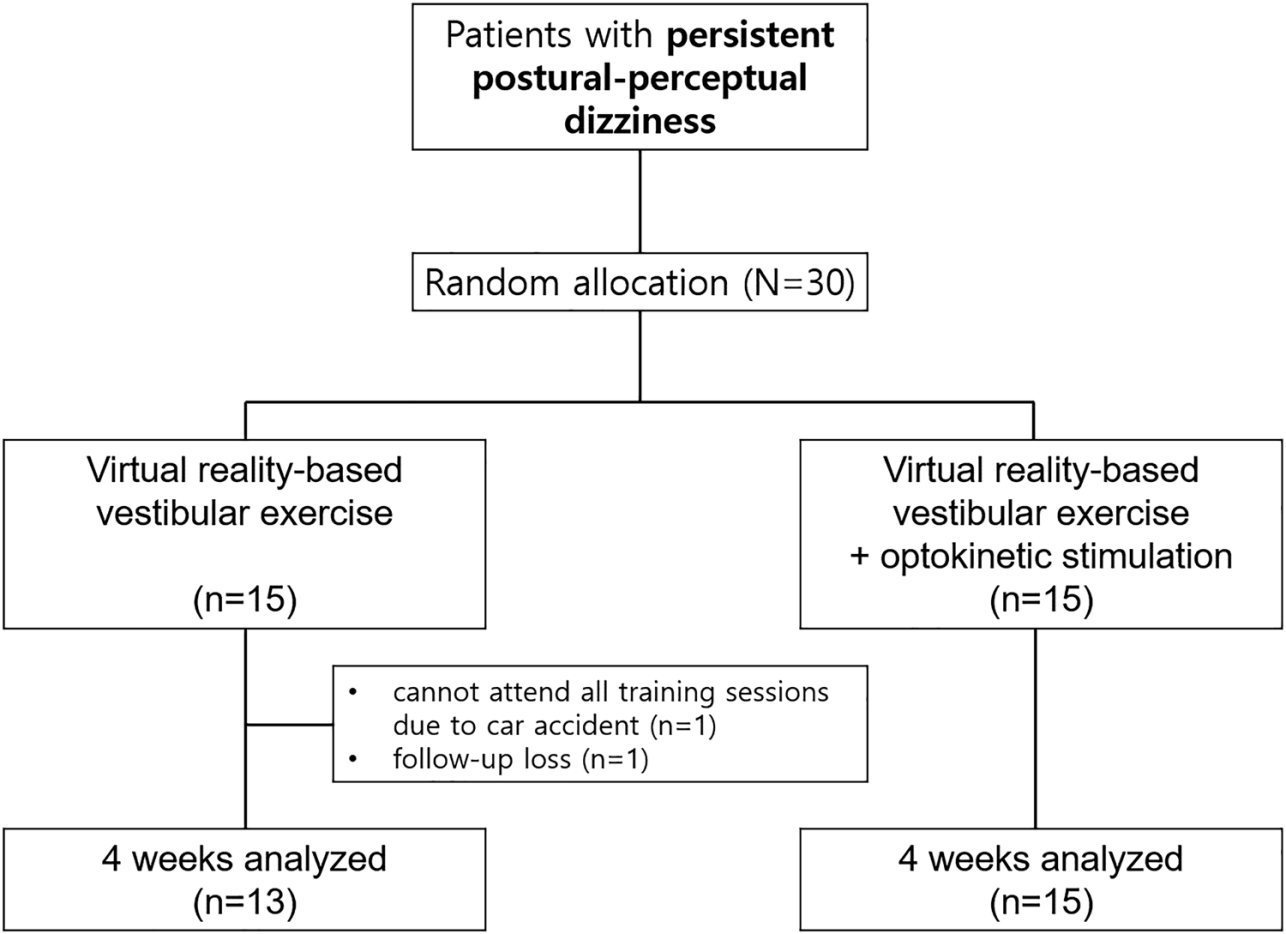
Consort diagram.

Of the 30 patients enrolled in this study, two patients in the VE group could not attend all training sessions (Fig. 1). Finally, 28 patients (age = median, 74.5, IQR 66–78, men = 12) completed the randomized intervention. There were no significant differences in the demographic characteristics, questionnaire scores, or timed up-to-go-test (TUG) time between the VE and VE + OS groups at baseline (Table 1).

**Table 1:** Median scores for outcome measures at baseline and 4 weeks in the vestibular exercise group and the vestibular exercise with additional optokinetic stimuli group.

	Baseline			After treatment		
	Vestibular exercise group (n = 13)	Vestibular exercise + optokinetic stimulation group (n = 15)	*p* value	Vestibular exercise group (n = 13)	Vestibular exercise + optokinetic stimulation group (n = 15)	*p* value
**Demographic characteristics**						
Age, year	75 [67,78]	71.5 [65,78]	0.555			
Sex, men/women	5/8	7/8	0.832			
**Questionnaires**						
Dizziness handicap inventory	42 (26–58)	34 (21–50)	0.413	26 (12–42)	28 (25–50)	0.387
Vestibular activities of daily living	65 (54–72)	55 (41–63)	0.156	50 (42–56)	53 (36–57)	0.786
Visual vertigo analogue scale	16 (10–38)	9 (6–21)	0.261	4 (4–10)	9 (4–13)	0.650
Beck's anxiety index	10 (5–15)	6 (2–12.5)	0.294	5 (3–11)	5 (1–12)	0.650
**Functional gait analysis**						
Timed up-to-go test, seconds	12 (10–14)	11 (9–15)	0.614	10 (9–12)	9 (8–12)	0.614
**Dynamic posturography**						
SOT1	92.5 (91–94.3)	93 (88.3–94.8)	0.776	92.5 (90.3–93.8)	92.5 (91.1–93.5)	0.934
SOT2	89 (83.3–91)	89.8 (88.5–91.9)	0.501	88.5 (83.8–89.8)	88.3 (88–90.5)	0.410
SOT3	88.5 (87.4–90.8)	88.5 (84.1–91.2)	0.979	89 (88.3–91.7)	88.5 (85–90.2)	0.208
SOT4	76.3 (73.7–78.3)	76.7 (72.8–82.3)	0.700	77 (74.2–81.7)	78.7 (75.2–80.9)	0.742
SOT5	59 (52.2–64.3)	59.5 (50–65.6)	1.000	67 (54.8–69)	64 (59.5–68.2)	0.661
SOT6	59.3 (46.4–66.7)	56.8 (50.1–61.6)	0.959	65.3 (52.7–67.5)	60.5 (49.3–66.7)	0.621
Composition	73 (68.5–75.5)	74 (68.3–76.8)	0.699	77 (71–79)	76 (70.3–77)	0.394

### Questionnaire scores and timed up-to-go test time

**Figure 2:**
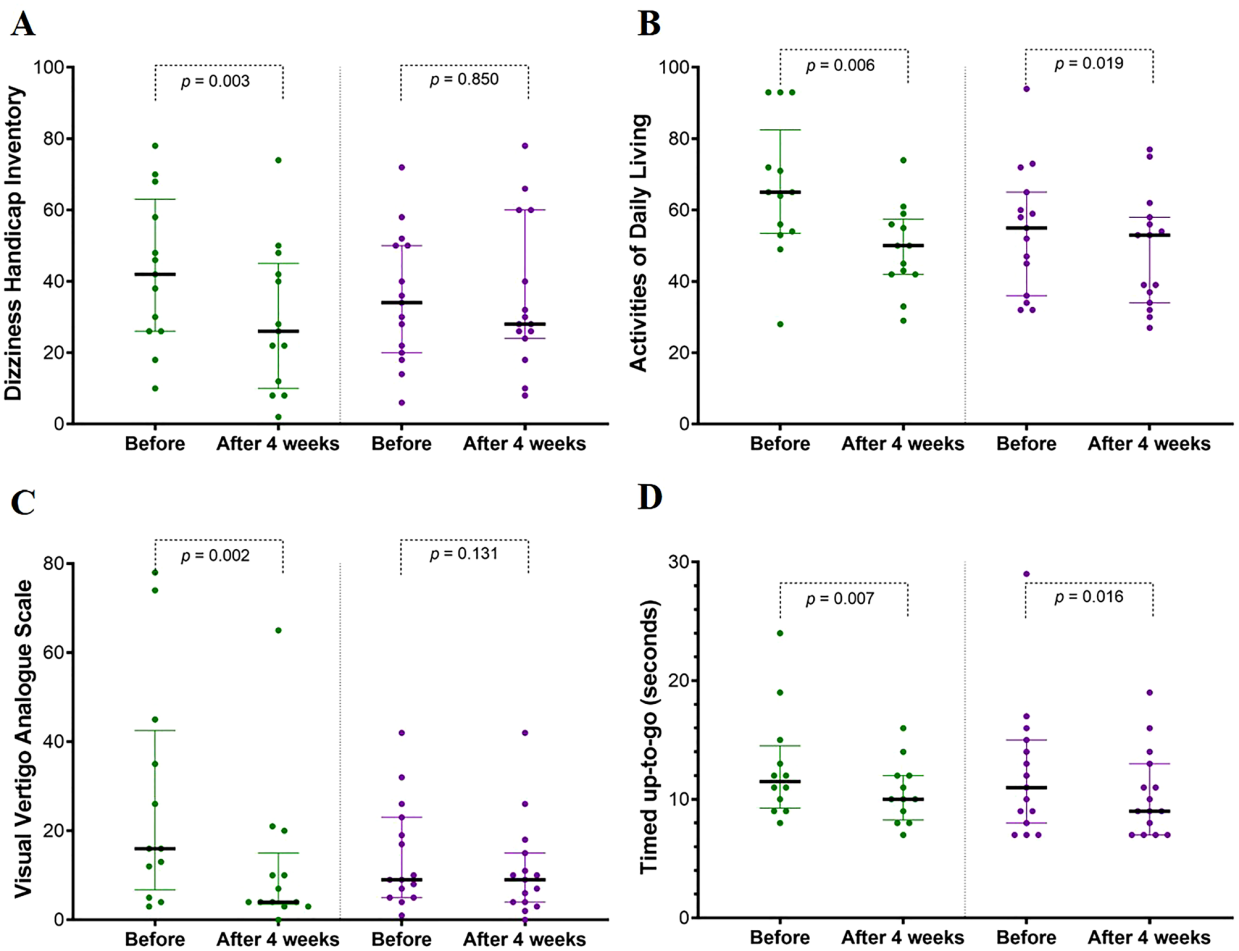
Dizziness handicap inventory (DHI, **A**), vestibular activities of daily living (ADL, **B**), visual vertigo analogue scale (VVAS, **C**), and time of timed up-to-go test (TUG, **D**) before and after treatment. All questionnaires and TUG time scores were significantly improved in the vestibular exercise (VE) group (green); however, only ADL and TUG time were improved in the VE with optokinetic stimulation (OS) group (purple). Every dot is each score or time of patient. The green or purple bars indicate the interquartile range, and the horizontal black bars identify the median value of each group. All *p* values were assessed with the Wilcoxon signed rank test.

From baseline to 4 weeks, the dizziness handicap inventory (DHI), activities of daily living (ADL), visual vertigo analogue scale (VVAS), and TUG time significantly improved in the VE group, while in the VE + OS group, only ADL and TUG time improved (Fig. 2). Between the two groups, DHI, ADL, VVAS, and TUG time did not differ after the intervention (Table 1).

### Dynamic posturography

The sensory organization test (SOT) of six conditions on dynamic posturography did not differ at baseline between the VE and VE + OS groups or did not significantly change after the intervention in either group (Table 1). No significant correlations were noted between each posturography score and VVAS at any assessment.

### Analysis of the visual vertigo analogue scale

**Figure 3:**
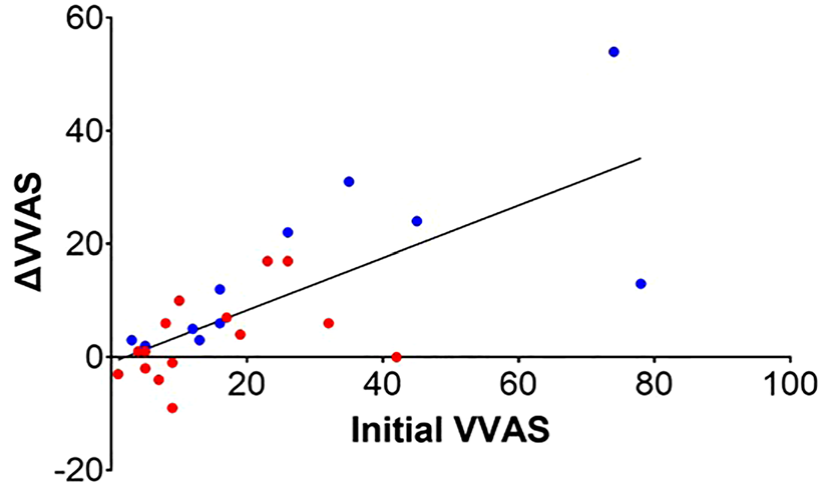
The change in VVAS after the 4-week program (ΔVVAS = VVAS at baseline—after 4 weeks, a negative value indicates aggravated visual vertigo) was correlated with VVAS at baseline in the VE group (blue dots, Spearman’s ρ = 0.881, *p* < 0.001) and the VE + OS group (red dots, Spearman’s ρ = 0.544, *p* = 0.036). The change in the VVAS score of all participants was correlated with the initial visual vertigo symptom (Pearson’s ρ = 0.716, *p* < 0.001, R^2^ = 0.513).

Although complex visual stimuli, including VRT, are helpful for patients with visual vertigo symptoms^8^, the association between the severity of visual vertigo symptoms and the effectiveness of visual stimuli has not been elucidated. We analysed the correlation between VVAS at baseline and the change in the VVAS score from baseline to 4 weeks in both groups to verify whether the initial severity of visual vertigo symptoms affected the final visual vertigo symptoms. The change in VVAS after completion of 4 weeks of exercise was significantly correlated with the initial VVAS in both the VE (Spearman’s ρ = 0.881, *p* < 0.001) and VE + OS groups (Spearman’s ρ = 0.544, *p* = 0.036, Fig. 3). Therefore, the change in the VVAS score of all participants was correlated with the initial visual vertigo symptom (Pearson’s ρ = 0.716, *p* < 0.001, R^2^ = 0.513).

### Simulator sickness

Three patients (10%) reported a slight intensity of stimulation sickness on one question of the simulator sickness questionnaire (SSQ), fatigue of the eye during VE.

## Discussion

To our knowledge, this study is the first randomized trial utilizing virtual reality-based VRT for the treatment of PPPD. We found that virtual reality-based VRT can improve vestibular symptoms, quality of life, and gait function in patients with PPPD. In both groups, patients with severe visual vertigo showed more significant improvements after completing our program than those with mild visual vertigo.

Sufficient evidence indicates that VRT is a safe and effective treatment for acute and chronic peripheral vestibulopathy^9,10^. Gaze stability exercises and complex visual stimuli exposure have already been verified and recommended as rehabilitation for patients with chronic peripheral vestibulopathy^9^; however, VRT should be carefully tailored to the needs of patients with PPPD^3^. Every day active sports can improve balance function at a young age, even for those with PPPD^11^, but the individual VRT for promoting habituation has to be attempted by stage because the phase approach is favourable to decrease patients’ high anxiety and excessive vigilance to balance sensation, especially in the elderly^3^. Although VRT has been proven to improve subjective symptoms and posturographic results in chronic subjective dizziness^12,13^, which type of exercise is effective specifically for PPPD has not been clarified.

Interestingly, gaze stability exercises can improve the subjective and behavioural performance of patients with unilateral vestibulopathy despite an absent change in VOR gain from semicircular canals^15^. The remaining otolith function^15^ and improvement of dynamic visual acuity^16^ were suggested as possible mechanisms. Likewise, repetitive head and neck movements, including gaze stability exercises, are regarded as effective methods to decrease sensitivity to one’s own movements (adaptation) and substitute other sensory systems for dynamic visual acuity, not restore vestibular loss in PPPD^3,5^.

Our program was designed to focus on (1) correct head and neck motion during gaze exercise movements based on vestibular adaptation according to the individual’s ability, and (2) adequate visual stimuli that were closest to reality and could arouse interest in promoting habituation and concentration. Because elderly individuals with chronic dizziness have a risk of falling, especially when unaccompanied by a supervisor, we did not include gait training in our exercise program.

Visual stimulation with optokinetic or complex backgrounds would be effective for the improvement of visual vertigo^7^. In a retrospective survey study, patients with PPPD answered that habituation exercise was effective for the relief of their symptoms^14^. VRT using complex visual stimuli for visual vertigo has been developed from a simple optokinetic drum and rotating chair to treadmill in a virtual grocery store on a screen^7,8,17^. However, although optokinetic stimuli are effective in chronic peripheral vestibulopathy regardless of their frequency, velocity, type of texture, and position^7^, they can be intolerable because optokinetic stimuli that are too intensive and frequent are nauseogenic^18^.

Optokinetic stimuli using a virtual reality system did not show additional efficacy in our study. Because the realistic background and/or active head-eye movements in our virtual reality-based VRT may fully satisfy the habituation process in the central nervous system of patients with PPPD^5^, additional optokinetic visual stimuli would be a surplus. The optimal type or feature of visual stimuli has not been settled for VRT, and a visual background such as a crowded square shown on the screen can be sufficient to improve visual vertigo symptoms in patients with unilateral vestibulopathy^8^. Moreover, marked improvement in our patients with severe visual vertigo reflects that the properties of visual stimuli should be individualized depending on the severity of symptoms in PPPD. Although visual vertigo is closely related to the main symptoms of PPPD, the quantity of dizziness from complex visual stimuli should be considered to develop a VRT strategy for PPPD.

Some studies have shown significant improvement of subjective symptoms or static posturographic assessment by VRT using virtual reality programs in patients with peripheral vestibulopathy or visual vertigo^8,19^. These studies used variable devices providing virtual reality situations from small and flat monitors to a planetarium with a treadmill. We designed our program to take wireless head mount displays because it does not take up much space, is relatively inexpensive we can operate it more easily and simply than projectors, screens, monitors, or other commercial rehabilitation tools (ex. Balance Rehabilitation Unit or Nintendo Wii), and it can produce very realistic stimuli in comparison to other bulky machines. Because enhanced reality and a high degree of presence can outperform the efficacy of VRT using a virtual reality system, HMD with a well-crafted exercise program would be the best choice for VRT, especially for inducing habituation and motivation in patients with visual vertigo^8^. Although the SOT of dynamic posturography did not change after our VRT, TUG was significantly improved. The gaze stabilization exercise, the main therapy in our system, can help elderly individuals with dizziness improve their gait function through adaptation of the vestibulo-ocular reflex, even in the absence of any vestibular dysfunction^16^. Additionally, sufficient habituation depending on the idiosyncratic strategies using these state-of-the-art facilities can be drawn to recover balance function during gait^6,19^.

The conflict between accommodation and vergence depth cues on stereoscopic displays or complex visual perception without self-motion could lead to significant motion sickness or discomfort when patients are exposed to complex virtual reality environments for a long time^18,20^. However, therapies using more complicatedly designed virtual reality are effective for anxiety, provoking realistic reactions to feared stimuli and dementia^18^. In elderly patients (n = 236) without any experience with the virtual reality system, virtual reality cognitive therapy was significantly effective in their depressive mood, but only 1.4% of patients reported severe simulator sickness^21^. Because our virtual reality program did not include the targets or background objects moving backward and forward, none of our patients complained of severe simulator sickness.

This study has several limitations. First, the duration of VRT and number of sessions might not be enough to improve patients’ symptoms. Because patients enrolled in our study were mostly elderly, they could not visit the hospital easily or frequently. Home-based exercise using our system would be attempted clinically and be more effective for patients than hospital-based exercise. Second, there was no improvement in the SOT of dynamic posturography. The relatively short study duration and lack of balance or gait training in our VRT program may have affected the posturographic results. Additionally, the initial SOT of our patients was not severe because we excluded patients with falling risk.

Our VRT program using a virtual reality system promoting vestibular adaptation and habituation can improve dizziness, quality of life, and gait function in patients with PPPD. Additional visual stimulation using optokinetic stimuli should be carefully provided depending on the quantity of visual symptoms.

## Methods

### Standard protocol approvals, registrations, and patient consent

The trial was registered at cris.nih.go.kr (KCT0003864, the date of first registration; 24/04/2019). This study was performed in accordance with ethical principles consistent with the Declaration of Helsinki. All of study protocol and informed consent were reviewed and approved by the corresponding health authorities and ethics boards/institutional review boards of Pusan National University Hospital (1903-001-076). Enrolled patients gave written informed consent for participation in the trial.

### Subjects

Between April 2019 and April 2020, 30 consecutive patients with a diagnosis of PPPD were recruited from a referred hospital. Subjects were between 51 and 82 years of age (mean ± SD = 71.3 ± 7.8). All participants fulfilled the diagnostic criteria of PPPD^2^. Patients were excluded if they (1) could not stand alone due to severe imbalance, neurologic deficit, or orthopaedic problem, (2) could not follow instructions of the rehabilitation program due to their cognitive problem, (3) were uncompensated peripheral vestibular loss, in which their dizziness or unsteadiness were provoked as distinct episodes of head motion-induced or physical examination including head thrust, headshake, or stepping tests^3^, or (4) had previous central vestibulopathy including stroke, infection, or demyelinating diseases, or vestibular migraine.

### Study design and randomization

We conducted a prospective randomized controlled study to determine the 4-week therapeutic efficacy of VE and VE + OS using a virtual reality system (Fig. 1). Based on the data from previous studies in chronic subjective dizziness^13^, we estimated that the proportion of 4-week efficacy of vestibular rehabilitation in chronic subjective dizziness would be 80% in VE and 40% in VE + OS. By adopting 0.9 power to detect a significant difference (*p* = 0.05, two-sided) and a dropout rate of 20%, 15 patients were required for each treatment arm.

Thirty patients were randomly assigned to the VE or VE + OS groups. The randomization procedure designed in permuted sizes of 4 blocks was based on the web-based program provided by REDCap© (Vanderbilt University, USA).

A physiotherapist conducted the disposed program approximately 20 min weekly for 4 weeks. The patients in the VE + OS group additionally had a crafted OS for 9 min. Before exercise on the visit day and after 4 weeks, we consecutively assessed the questionnaires, TUG test, and Computerized Dynamic Posturography in all participants. The participants also answered questions about simulator sickness after 4 weeks.

### Intervention program

#### Virtual reality equipment

An OCULUS GO virtual reality headset and controller were used for our program. The head mount display (HMD) is mobile and wireless and has a field of view of 110 degrees. The resolution was 2560 × 1440 K, and the refresh rate was 60 Hz. The total hardware weight of hardware was 468 g.

#### Vestibular exercise program using virtual reality

We developed VE and OS with a virtual reality system and applied a patent collaborating the manufacturer (JCS Co., Ltd.). The exercise protocol was described in Table 2. In a virtual reality environment, the target looks like a robot and moves horizontally and vertically one metre ahead along the centre. The target guided the participants to follow and let them know whether they were exercising exactly 15 degrees around the centre by audio guidance. When the participants successfully completed the exercise 10 times, the speed of the exercise was increased. The optokinetic stimulation program consisted of stars rotating around the yaw, pitch, and roll axes approximately 3 min in each axis in the night sky (Video 1).

**Table 2:** Vestibular exercise customized on adaptation and habituation.

	Exercises	Background	Direction of the target	Frequency of head movements	Duration (min)
Session 1: Adaptation with stable background	Vestibulo-ocular reflex exercise: the patients rotate their head 10 times 15° around the target* on the centre along the pitch and yaw axis	Static environment: a living room Dynamic environment: under the sea, with fish	No movement	0.5–1 Hz	5–7
Session 2: Adaptation with habituative background	Visual guided vestibulo-ocular reflex exercise: the patients follow the head AND eye to the target*. The target* moves 15° horizontally and vertically	Static environment: a living room Dynamic environment: under the sea, with fish	Horizontal and vertical	0.5–1 Hz	5–7
Session 3: Adaptation	Active head and eye exercise: the patients rotate their head and shift their gaze rapidly to catch-up to the target (spacecraft), which randomly appears every 10 s and moves around within a 270° visual field	The night sky (black space)	Random, within a 270° visual field	0.5–1 Hz	5–7
Optokinetic stimulation: Additional habituation	The patients watch many stars in the night sky, which rotate counter-clockwise along the pitch, yaw, and roll axis of their head	The night sky (black space)	Counter-clockwise along the pitch, yaw, and roll axis of the patient’s head	5–10°/seconds	9

The target* in sessions 1 and 2 looks like a robot, which can move horizontally and vertically, one metre ahead along the centre. The target guided the participants to follow and let them know whether they were exercising exactly 15 degrees around the centre. When the participants successfully completed the exercise 10 times, they could increase the exercise.

### Outcome measures

#### Questionnaires

- The DHI contains 25 questions designed to assess a patient’s functional, emotional, and physical symptoms that is validated in Koreans. A score of 0 indicates no discomfort, and a score of 100 indicates a significant self-perceived handicap^22^.
- The ADL contains 28 self-rated questions for measuring the quality of life, which is scaled 10 points and divided into functional, ambulation, and instrumental skills for determining the level of functional limitation or disability in people with vestibular disorders^23^.
- The VVAS consists of nine visual analogue scales, and subjects were asked to rate no dizziness (0) to extreme dizziness (10). Each scale pertains to a specific visual vertigo provoking situation (walking into supermarket, riding in a car, under florescent lights, at an intersection, being in shopping centres, riding on escalators, watching movies at a theatre, on a patterned floor, and watching television)^24^.
- The Beck Anxiety Inventory is a 21-item self-report measure of the level of anxiety a person has experienced during the previous week.

#### Timed up-to-go test

The participants were instructed to get up from a chair, walk three metres to a wall in an unobstructed room or hall, turn around, walk back, and sit down again. The time (seconds) taken to complete the test was recorded before and after 4 weeks of treatment^25^.

#### Computerized dynamic posturography

A computerized dynamic posturography system using the SOT protocol (NeuroCom Smart Equitest system) was performed before and after the intervention. The subject stands on a computerized platform in six conditions (eyes open, eyes closed, surrounding walls moving, platform surface moving, platform surface moving with eyes closed, and walls and platform surface moving together). Three trials are performed in each condition. The computerized platform measures and calculates the subject’s postural stability based on the subject’s input through the platform force plates during the various conditions. If a patient is unable to complete a trial during the test, it is marked as a “fall,” resulting in a score of 0.

### Simulator sickness questionnaire

The SSQ was used to assess physical discomfort after exposure to the virtual reality program. The SSQ contains 16 items to measure fatigue, headache, eye strain, difficulty focusing, increased salivation, sweating, feeling of nausea, difficulty concentrating, fullness of the head, blurred vision, dizziness with eyes open/closed, loss of orientation with respect to vertical upright, and general discomfort. The intensity of sickness was measured at four levels: severe, moderate, slight, and none^18^.

### Statistical analysis

The Mann–Whitney U-test and Wilcoxon signed rank test were used to compare the continuous variables, including age, all questionnaires, TUG time, and results from dynamic posturography. Fisher’s exact test was applied for the categorical variables. The relationships between the change in VVAS and VVAS at baseline were assessed with the Spearman or Pearson correlation. All statistical procedures were performed using SPSS statistical software (version 23.0; SPSS, Chicago, IL, USA), and *p* < 0.05 was considered significant.

### Ethical standard

This study followed the tenets of the Declaration of Helsinki and was performed according to the guidelines of the Institutional Review Board of Pusan National University Hospital (1903-001-076).

## Supplementary Information

Supplementary Video.

Supplementary video caption.

## Data Availability

Anonymized data will be shared by request from any qualified investigator.
