# Supplementary material for: Effect of vestibular exercise and optokinetic stimulation using virtual reality in persistent postural-perceptual dizziness
Source: Sci Rep. 2021 Jul 14;11:14437. doi: 10.1038/s41598-021-93940-z (PMC8280184; doi:10.1038/s41598-021-93940-z)
Supplement: Supplementary file 2 — Supplementary video caption. [file 41598_2021_93940_MOESM2_ESM.docx]

**Video Legend**

Video 1. The vestibular exercise program consists of three sessions. The first session is an adaptative vestibule-ocular reflex exercise in a stable background, which is a fixed target in a room. The patient with a head mount display had to rotate over 15 degrees around the target. The second session is an adaptive visual-guided vestibulo-ocular reflex exercise with a habituative background (fish in the ocean). The third session is an active head and neck exercise. The patient has to catch spacecrafts that appear suddenly in the patient’s visual field. Because the field is 270 degrees based on the centre, the patients should rotate their head quickly to find the target. Every session is repeated with increasing difficulty to move the head and/or the eyes faster. Additional optokinetic stimulation using small lightening dots like stars in the night sky rotate clockwise around the patient. The subject in the right corner of the screen is the first author of this study.
